# Supplementary material for: A warm jet in a cold ocean
Source: Nat Commun. 2021 Apr 23;12:2418. doi: 10.1038/s41467-021-22505-5 (PMC8065036; doi:10.1038/s41467-021-22505-5)
Supplement: Supplementary file 1 — Supplementary Information [file 41467_2021_22505_MOESM1_ESM.pdf]

Supplementary information for 'A warm jet in a cold ocean'

Jennifer A. MacKinnon, Harper L. Simmons, John Hargrove, Jim Thomson, Tom Peacock, Matthew H. Alford, Benjamin I. Barton, Samuel Boury, Samuel D. Brenner, Nicole Couto, Seth L. Danielson, Elizabeth C. Fine, Hans C. Graber, John Guthrie, Joanne E. Hopkins, Steven R. Jayne,Chanhyung Jeon, Thilo Klenz, Craig Lee, Yueng-Djern Lenn, Andrew J. Lucas, Björn Lund, Claire Mahaffey, Louisa Norman, Luc Rainville, Madison M. Smith,Leif N.Thomas, Sinhué Torres-Valdés, Kevin R. Wood

March 7, 2021

Water mass definitions

Different components of Pacific Origin water arriving in the Canada Basin are often given different names that reflect their unique histories and trajectories within the basin. Most commonly, PSW is commonly divided into the warmest, freshest water known as Alaska Coastal Water (ACW), and a cooler slightly saltier variety known as Bering Sea Water (BSW) (1; 2). ACW flows along the eastern side of the Bering and Chukchi seas as a coastal current, and experiences significant river input (1). BSW is found in the central Chukchi shelf, and may enter the Canada Basin though Ekman pumping at the shelbreak jet (3), or may turn eastward near the shelfbreak edge to join ACW flowing through Barrow Canyon (4). Older definitions describe ACW and BSW as having salinities of $31 \leq S \leq 32$ and $32 \leq S \leq 33$ respectively (1; 2). Recently these water masses, particularly the warmest ACW, have been freshening, likely due in part to increased glacial ablation in the Gulf of Alaska (5). As a result, more recent studies have found the need to adjust water mass definitions to include fresher water (3). At the risk of glossing over these differences in pathways, for simplicity we use the 23.2 $\mathrm{kg\,m^{-3}}$ and 25.2 $\mathrm{kg\,m^{-3}}$ isopycnals as boundaries for the broader PSW class, as they bracket the specific temperature peak observed here. We use the PSW term generally to be inclusive of a broad audience, though majority of the warm water seen here is most consistent with ACW.

Balance and centrifugal instability

In the main text we comment on the importance of flow curvature in thermal wind balance. Traditionally thermal wind is written as a balance between the vertical derivatives of the Coriolis force acting on the along-jet flow and the cross-jet pressure gradient (6). Using the traditional definition, the vertical shear in the primary cross-jet section is significantly larger than could be supported by lateral buoyancy gradients, for both positive and negative values (Fig. S1, left). One possible explanation is that the section here was not perfectly aligned with the true cross-jet reference frame. However in order for that to explain an observed discrepancy of $\sim 50\%$, the section orientation would need to be off by 25 degrees, which seems inconsistent with the jet velocity vectors pictured in Figure 1c of the main text. A more likely explanation lies in the curvature visible in this meandering jet. For curved flows that circulate anticyclonically (clockwise in the Northern Hemisphere), the Coriolis force acting on the along-jet flow must be large enough to balance not only the lateral pressure gradients but also supply the centripetal acceleration associated with this meander (7).

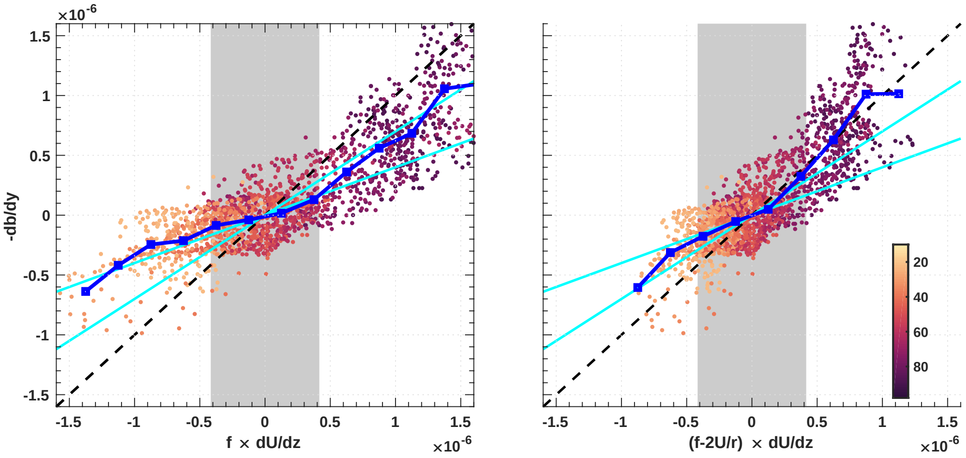

Figure S1: Testing thermal wind balance. Left: scatter plot of the vertical gradient of along-jet velocity (x-axis here, panel (e) in Figure 2 of the main text, and cross-jet buoyancy gradient (y-axis here, panel (f) in Figure 2 of the main text). Data shown is between 20 and 90 m depth, and cross-front distances less than 12 km. Color indicates depth below the surface, in m. The dark blue markers/line are bin averages. The dotted black line is a one-to-one slope. The two cyan lines indicate slopes of 0.7 and 0.4 respectively. The grey shaded area indicates the uncertainty in the shear measurement. Right: similar, but now including the additional force required needed for centripetal acceleration of this curving jet.

Inclusion of this term produces a closer agreement between flow speed and lateral density gradients following a force balance known as the gradient wind balance (8) (Fig. S1, right).

Having said this, at depths near 80 m where the lower front is located, the gradient wind balance breaks down (e.g. Fig. S1 right, brown points) in the sense to drive an ageostrophic circulation that would flatten out isopycnals similar to the secondary circulation inferred from the omega-equation. This force imbalance could be a signature of centrifugal instability (CI), since it is expected that the gradient wind balance breaks down as a centrifugally unstable flow adjusts to equilibrium, resulting in an acceleration of the ageostrophic circulation. The strength of this circulation can be estimated from the gradient wind imbalance which is around 5×10^{-7} s^{-2} near the lower front. If this imbalance were to accelerate the ageostrophic circulation over a time scale $1/f$ (i.e. a typical time scale of evolution for CI), then the ageostrophic shear that would result would be 5×10^{-3} s^{-1}. This can be compared to the ageostrophic shear inferred from the omega-equation by taking the second derivative with respect to z of the streamfunction in Fig 4e, which near the lower front yields a shear of 1×10^{-4} s^{-1}, over an order of magnitude weaker. This suggests that adjustments towards equilibrium associated with the submesoscale dynamics of CI result in stronger ageostrophic circulations than strain-driven overturning motions as inferred from the omega equation.

Previous work has discussed related conditions for CI associated with flow separation from or interaction with topography, both in this region (9) and elsewhere (10; 11). Qualitatively, simulations of CI show that that the instability tends to redistribute momentum in the cross-jet direction, through a convoluted pattern of filaments. The instability has a complex three-dimensional structure, but the net effect is to spread out momentum until the negative vorticity is restored to stable values ($Ro > -1$). In that sense its net expected effect is not dissimilar to the qualitative pattern of cross-jet flow shown in Figure 4e. An important difference is that while the omega equation is based upon the theory of frontogenesis, which assumes the weak overturning circulation evolves slowly in time and maintains the thermal wind balance, CI is expected to create much larger cross-jet veloc-

ities, not follow a force balance, and exhibit strong time dependence. Indeed, as described above, there are certain locations on the section where the thermal wind balance breaks down in the sense to drive an overturning circulation that flattens isopcynals and could be a signature of CI.

Coincident with curved scallops of cooler, freshwater water, lateral stirring also brings in water with higher total potential vorticity from outside the jet (Fig. 4d). This type of intermixing and homogenization of PV between the jet and background water (particularly the relative vorticity component) is also qualitatively consistent with theoretical and numerical studies of CI (12). The result of this mixing process would be eddies with Ro > -1 carrying diluted PSW, consistent with the observed bulk values of the vorticity in Figure 2g and the evolution of the T-S relation seen in Figure 3.

Ekman and turbulent thermal wind transport of near-surface water

Water in the surface boundary layer also experiences direct wind and wave forcing that partially decouple it from the more stratified ocean below. During this survey, the measured wind is from the East. In the upper few meters, wind stress likely pushes water directly downwind through Stokes drift (13; 14; 15).Averaged over the surface mixed layer, wind-driven Ekman transport leads to water movement to the right of the wind. For this Easterly wind, the sense of Ekman transport would be to push near-surface freshwater to the right/northward in Figure 2 of the main paper, which is opposite to the visual suggestion of freshwater moving left to cover subducting PSW. At the same time, in a turbulent surface layer, the Coriolis force acts on vertical diffusion of the strongly sheared geostrophic currents shown in Figure 2 of the main paper. This turbulent thermal wind (TTW) tends to spread light freshwater filaments out sideways in both directions, with velocities that can be larger than that of the wind-driven Ekman transports (16).

The turbulent thermal wind velocity (Fig. S2) is calculated as $v_{TTW} = -(d_E/2)(dU_g/dz)$ where $d_E = \sqrt{2\nu/f}$ is the Ekman depth, ν the turbulent viscosity in the surface layer, and $dU_g/dz = -(1/f)(db/dy)$ is the geostrophic shear (16). The Ekman depth roughly represents the depth over which wind momentum is mixed. Here we set d_E =5 m, based upon inspection of velocity profiles from nearby SWIFT drifters, which show uniform velocity with depth for at least the top 5 meters. The Ekman velocity is calculated as $v_{Ek} = -\tau/[f\rho_0 d_E]$, where τ is the component of the wind stress perpendicular to this section.

The rough magnitude of the expected average Ekman and TTW velocities are plotted in Figure S2c (red and blue respectively). Relative vorticity and flow curvature may also affect Ekman transport, but are not shown here (17; 18; 19). Where near-surface density is at a local minimum (Fig. S2b, blue), TTW velocities spreading this freshwater both southward (on the left of density minima) and northward (to the right of density minima) are often larger than those of Ekman velocities (Fig.S2c, blue). The combination of mesoscale stirring and TTW applied to near-surface freshwater filaments thus tends to evenly spread light, fresh surface waters over this region.

The major caveat of this calculation is the use of a single Ekman depth, which is not consistent with the observation that the near-surface layer varies in thickness from 5 to 15 m (Fig. S2a). However, the calculation here is meant only to highlight the often differing directions of Ekman and TTW transport, and point to the often larger contribution of TTW. This particular choice of d_E gives a conservative lower bound to v_{TTW}; if the real Ekman depth were larger at any point along this section, the associated TTW currents would be as well.

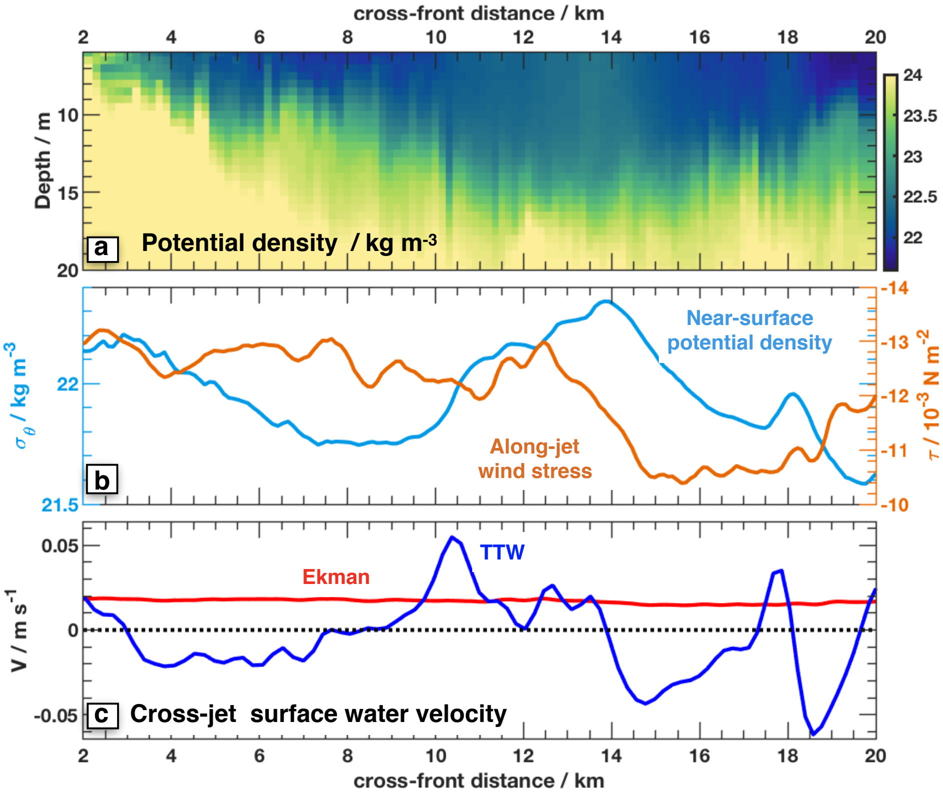

Figure S2: Cross-jet near-surface freshwater transport estimates.a) near surface potential density for the same section shown in Figure 2 of the main text. b) the along-front component of wind stress (orange, negative values indicate an Easterly wind), and the nearest surface measurement of potential density (blue). c) cross-jet velocity expected for surface water from linear Ekman transport (red), and turbulent thermal wind (blue).

Surface heat fluxes

Net air-sea heat flux (positive indicating heat flux *into* the ocean) has four components: net short and long wave radiation, sensible and latent heat loss. Net longwave heat flux into the ocean is computed using established algorithms (20). Net shortwave heat is computed from the measured downwelling shortwave radiation with albedo taken from Payne (21). Turbulent heat sensible and latent fluxes are computed using the COARE 3.5 algorithm (22) with modification based on the CLIMODE, MBL and CBLAST experiments (23). Winds were taken from the ship's forward anemometer. Anemometers and RH on the *Sikuliaq* are 16.0m and were adjusted to the standard 10m height expected by the COARE algorithm. Values for relative wind directions greater than 60 degrees from the stern redacted. Surface heat fluxes were only computed in open water and used the radiometric skin temperature rather than the 6 meter thermosalinograph temperature. Open water was defined as times when the skin temperatures was above the freezing point of seawater computed using the measured skin temperature and the thermosalinograph salinity; data gaps in the third and fourth panels reflect times when the ship was in the ice pack.

The net short and long wave radiation have a strong diurnal cycle (Fig. S3b). Sensible and latent heat fluxes are more irregular, reflecting the changing air and sea heat conditions over the course of this survey (Fig. S3c). During the Sept 15-17 ship survey, surface temperatures were elevated and were warmer than surface air temperature, producing a -122 $\mathrm{Wm^{-2}}$ net heat loss from the ocean to the atmosphere (Fig. S3d). The September 24 survey occurred closer to neutral conditions with approximately -16 $\mathrm{Wm^{-2}}$ of cooling during the survey.

Turbulent mixing in the ocean interior

Turbulent heat fluxes within the ocean are calculated using data from the the Modular Microstructure Profiler (see Methods). An example average profile is shown in Figure S4. Within and below the peak of warm PSW, turbulent dissipation rate levels are modest, consistent with previous observations of weak turbulence in the Canada Basin (24; 25). Dissipation rate values steadily rise between 30 m and the surface. Very near the surface turbulence estimated from a nearby SWIFT drifter (see Methods) is several orders of magnitude larger, as typical of enhanced turbulence near the ocean surface. Diapycnal diffusivity calculated using a standard mixing efficiency of 0.2 (26; 27) is near molecular values below 30 m depth. Above 30 m the diffusivity also rises steadily. Calculated turbulent heat fluxes are weak below 30 m, but rise to 10-50 $\mathrm{Wm^{-2}}$ in the upper 20 m, carrying heat upward towards the surface. The strong near surface turbulence as measured by the SWIFT data suggest that any heat that is fluxed through the base of the surface mixed layer will quickly be conveyed to the ocean surface, connecting these results and the air-sea fluxes presented in Figure S3. Where diffusive convection is present, mixing efficiency is likely closer to unity, so that the the heat fluxes presented in Figure S4 may be biased low by up to a factor of 5 (28).

Biogeochemical properties

Seawater samples were collected during the 2018 R/V *Sikuliaq* cruise from Niskin bottles mounted on the CTD frame. These samples were collected on behalf of the NERC Changing Arctic Ocean's ARISE project that seeks to map Arctic isoscapes as biomarkers to signal changes in trophic levels over time (29). The locations of the CTD casts are presented in Table S1, and shown as white dots in Figure 3g of the main text. The results of the particulate carbon and nitrogen, and carbon and nitrogen isotopic analysis for the R/V *Sikuliaq* are presented in Table S2 below.

Cast number	Longitude	Latitude	Context
11	151.0748 W	72.2061 N	Within Jet
12	148.9888 W	71.8647 N	Within Jet
13	149.0202 W	71.9298 N	Within Jet
14	148.6755 W	73.0169 N	Outside Jet
15	148.9088 W	72.8771 N	Outside Jet
16	146.0197 W	73.0828 N	Outside Jet

Table S1: Location of CTD casts used for biogeochemical bottle samples presented here.

	δ^{15}N – PN (‰)	δ^{13}C – PC (‰)	PON (μgL^{-1})	PC (μgL^{-1})
**Within Jet**	6.7 ± 2	-26.2 ± 0.8	12.1 ± 5.5	62.0 ± 28.9
**Outside Jet**	4.6 ± 3.2	-28.8 ± 0.1	5.5 ± 0.5	25.6 ± 3.2

Table S2: Mean (± 1 standard deviation) of particulate organic nitrogen (PON), particulate carbon (PC), nitrogen (δ^{15}N-PN) and carbon isotope (δ^{13}C-PC) samples between the 1023.3-1025.2 kg m^{-3} isopycnals for CTD casts taken within and outside of the plume. The number of samples is n=3 for all data.

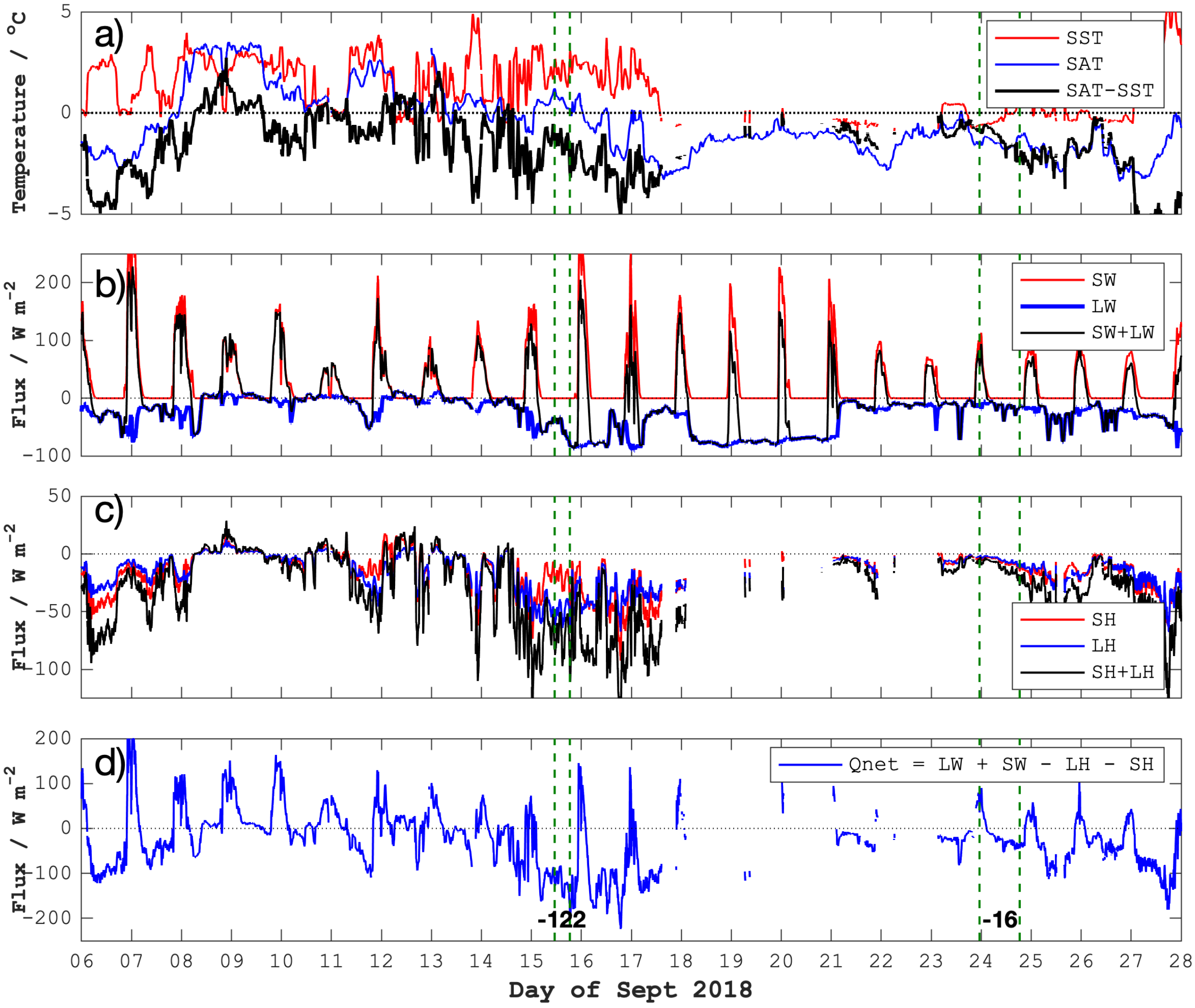

Figure S3: Ship-measured parameters relevant to air-sea heat fluxes, in panels of: a) surface sea and air temperatures, b) short- and long-wave radiative fluxes [W/m^2], c) sensible and latent turbulent fluxes [W/m^2], d) net surface heat flux. The Sept 15 and 24 survey periods are indicated as vertical dashed lines. During Sept 18-23 there were significant periods where turbulent fluxes could not be computed due to the presence of sea ice.

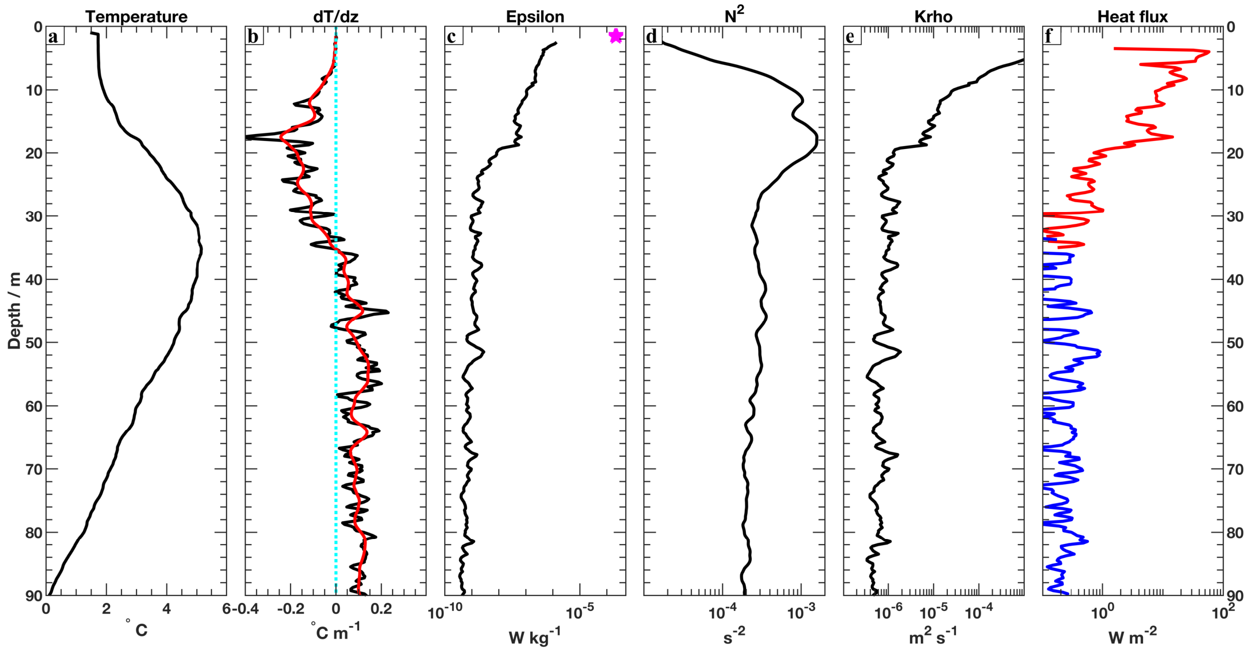

Figure S4: Microstructure profiles that go into heat flux calculations, from an average of 39 profiles taken between 21:11 on 16 Sept and 01:22 on 17 Sept, between latitudes of 71.8733N and 72.0059N along the MMP profiling track indicated in Figure 1c of the main text. Quantities include a) Temperature; b) vertical temperature gradient (black) and smoothed version (red), plus zero line; c) the dissipation rate of turbulent kinetic energy, ϵ from MMP (black) and a nearby SWIFT drifter (magenta star) d) Buoyancy frequency, N^2; e) Diapcynal diffusivity calculated using mixing efficiency of 0.2; f) turbulent heat flux, where red represents upward fluxes and blue downward

References

[1] Coachman, L. & Barnes, C. The contribution of Bering Sea water to the Arctic Ocean. *Arctic* **14**, 147–161 (1961).

[2] Steele, M. *et al.* Circulation of summer Pacific halocline water in the Arctic Ocean. *Journal of Geophysical Research: Oceans* **109** (2004).

[3] Timmermans, M.-L. *et al.* Mechanisms of Pacific Summer Water variability in the Arctic's Central Canada Basin. *Journal of Geophysical Research: Oceans* **119**, 7523–7548 (2014).

[4] Pickart, R. S. *et al.* Seasonal to mesoscale variability of water masses and atmospheric conditions in Barrow Canyon, Chukchi Sea. *Deep Sea Research II* **162**, 32–49 (2019).

[5] Danielson, S. *et al.* Manifestation and consequences of warming and altered heat fluxes over the Bering and Chukchi Sea continental shelves. *Deep-Sea Research II* (2020).

[6] Vallis, G. K. *Atmospheric and oceanic fluid dynamics* (Cambridge University Press, 2017).

[7] Shakespeare, C. J. Curved density fronts: Cyclogeostrophic adjustment and frontogenesis. *Journal of Physical Oceanography* **46**, 3193–3207 (2016).

[8] Holton, J. *An Introduction to Dynamic Meteorology* (Academic Press, San Diego, CA, United States, 1992).

[9] Spall, M. A., Pickart, R. S., Fratantoni, P. S. & Plueddemann, A. J. Western Arctic shelfbreak eddies: Formation and transport. *Journal of Physical Oceanography* **38**, 1644–1668 (2008).

[10] Gula, J., Molemaker, M. J. & McWilliams, J. C. Topographic generation of submesoscale centrifugal instability and energy dissipation. *Nature communications* **7**, 1–7 (2016).

[11] Wenegrat, J. O. & Thomas, L. N. Centrifugal and symmetric instability during Ekman adjustment of the bottom boundary layer. *Journal of Physical Oceanography* **50**, 1793–1812 (2020).

[12] Kloosterziel, R., Carnevale, G. & Orlandi, P. Inertial instability in rotating and stratified fluids: barotropic vortices. *Journal of Fluid Mechanics* **583**, 379 (2007).

[13] Rascle, N., Ardhuin, F. & Terray, E. A. Drift and mixing under the ocean surface: A coherent one-dimensional description with application to unstratified conditions. *Journal of Geophysical Research: Oceans* **111** (2006).

[14] Tamura, H., Miyazawa, Y. & Oey, L.-Y. The Stokes drift and wave induced-mass flux in the North Pacific. *Journal of Geophysical Research: Oceans* **117** (2012).

[15] Röhrs, J. & Christensen, K. H. Drift in the uppermost part of the ocean. *Geophysical Research Letters* **42**, 10–349 (2015).

[16] McWilliams, J. C. Submesoscale currents in the ocean. *Proceedings of the Royal Society A* **472**, 20160117 (2016).

[17] Stern, M. E. Interaction of a uniform wind stress with a geostrophic vortex. In *Deep Sea Research and Oceanographic Abstracts*, vol. 12, 355–367 (Elsevier, 1965).

[18] Mahadevan, A. & Tandon, A. An analysis of mechanisms for submesoscale vertical motion at ocean fronts. *Ocean Modelling* **14**, 241–256 (2006).

[19] Wenegrat, J. O. & Thomas, L. N. Ekman transport in balanced currents with curvature. *Journal of Physical Oceanography* **47**, 1189–1203 (2017).

[20] Dickey, T., Manov, D., Weller, R. & Siegel, D. Determination of longwave heat flux at the air-sea interface using measurements from buoy platforms. *Journal of Atmospheric and Oceanic Technology* **11**, 1057–1078 (1994).

[21] Payne, R. E. Albedo of the sea surface. *Journal of the Atmospheric Sciences* **29**, 959–970 (1972).

[22] Fairall, C. W., Bradley, E. F., Hare, J. E., Grachev, A. A. & Edson, J. B. Bulk parameterization of air-sea fluxes: Updates and verification for the COARE algorithm. *Journal of Climate* **16** (2003).

[23] Edson, J. B. *et al.* On the exchange of momentum over the open ocean. *Journal of Physical Oceanography* **43**, 1589–1610 (2013).

[24] Lincoln, B. J. *et al.* Wind-driven mixing at intermediate depths in an ice-free Arctic Ocean. *Geophysical Research Letters* **43**, 9749–9756 (2016).

[25] Shaw, W. J. & Stanton, T. P. Vertical diffusivity of the western Arctic Ocean halocline. *Journal of Geophysical Research* **119**, 5017–5038 (2014).

[26] Osborn, T. R. Estimates of the local rate of vertical diffusion from dissipation measurements. *J. Phys. Oceanogr.* **10**, 83–89 (1980).

[27] Gregg, M., D'Asaro, E., Riley, J. & Kunze, E. Mixing efficiency in the ocean. *Annual review of marine science* **10**, 443–473 (2018).

[28] Fine, E. C., MacKinnon, J. A., Alford, M. H. & Mickett, J. B. Microstructure observations of turbulent heat fluxes in a warm-core Canada Basin eddy. *Journal of Physical Oceanography* **48**, 2397–2418 (2018).

[29] de la Vega, C., Jeffreys, R. M., Tuerena, R., Ganeshram, R. & Mahaffey, C. Temporal and spatial trends in marine carbon isotopes in the Arctic Ocean and implications for food web studies. *Global Change Biology* **25**, 4116–4130 (2019).
